# “Salus Populi Suprema Lex”: Considerations on the Initial Response of the United Kingdom to the SARS-CoV-2 Pandemic

**DOI:** 10.3389/fpubh.2021.646285

**Published:** 2021-09-30

**Authors:** Evaldo Favi, Francesca Leonardis, Tommaso Maria Manzia, Roberta Angelico, Yousof Alalawi, Carlo Alfieri, Roberto Cacciola

**Affiliations:** ^1^Department of General Surgery, Renal Transplantation, Fondazione IRCCS Ca' Granda Ospedale Maggiore Policlinico, Milan, Italy; ^2^Department of Clinical Sciences and Community Health, University of Milan, Milan, Italy; ^3^Intensive Care Unit, Department of Surgical Sciences, Università di Tor Vergata, Rome, Italy; ^4^HPB Surgery and Transplantation, Department of Surgical SciencesUniversità di Tor Vergata, Rome, Italy; ^5^Department of Surgery, Kidney Transplantation, King Salman Armed Forces Hospital, Tabuk, Saudi Arabia; ^6^Department of Internal Medicine, Nephrology, Dialysis and Renal Transplantation, Fondazione IRCCS Ca' Granda Ospedale Maggiore Policlinico, Milan, Italy

**Keywords:** SARS-CoV-2, COVID-19, coronavirus, pandemic, clinical governance, non-pharmaceutical intervention, modelling, public health

## Abstract

In several countries worldwide, the initial response to coronavirus disease 2019 (COVID-19) has been heavily criticized by general public, media, and healthcare professionals, as well as being an acrimonious topic in the political debate. The present article elaborates on some aspects of the United Kingdom (UK) primary reaction to SARS-CoV-2 pandemic; specifically, from February to July 2020. The fact that the UK showed the highest mortality rate in Western Europe following the first wave of COVID-19 certainly has many contributing causes; each deserves an accurate analysis. We focused on three specific points that have been insofar not fully discussed in the UK and not very well known outside the British border: clinical governance, access to hospital care or intensive care unit, and implementation of non-pharmaceutical interventions. The considerations herein presented on these fundamental matters will likely contribute to a wider and positive discussion on public health, in the context of an unprecedented crisis.

## Introduction

“*Salus populi suprema lex”*: the quote from Cicero had undoubtedly a wider meaning, embracing welfare, justice, economy; beyond the actual health of the people.

Since the World Health Organization (WHO) declared the Coronavirus Disease 2019 (COVID-19) a pandemic on 11th March 2020 (1), all governments across the globe have adopted emergency legislations aimed to contain the impact of the virus. However, in several countries, the legislative effort and the stringent measures implemented were not spared by criticism on their efficacy and timing. In particular, one of the most debatable initial response to SARS-CoV-2 in Western Europe has occurred in the United Kingdom (UK).

In this article, we discuss some relevant aspects of the initial response (from February to July 2020) to the COVID-19 pandemic in the UK. Such aspects were not fully considered by the scientific community, as much as by the British and international Main Stream Media (MSM).

The domains we have identified for our considerations are: clinical governance, access to hospital and intensive care unit (ICU), non-pharmaceutical intervention (NPI), and modelling.

### Clinical Governance

Governance is “*de facto”* engraved in the professional duties of any clinical or academic practice. We all know how inconceivable it is in modern medicine suggesting an intervention, a clinical protocol or a research trial that is not supported by substantial scientific evidence. The very basis of patient safety was built on “*Primum non nocēre”*. This is not just a motto. It is a fundamental principle that protects who is vulnerable while guiding who is caring for them.

The unprecedented challenges posed by the first wave of COVID-19 found the global healthcare communities unprepared. In the UK, such unpreparedness revealed very deep fractures between the reality of the National Health Service (NHS) and the needs of both the population and healthcare professionals (2). Unexpectedly, the pandemic brought under public scrutiny the validity and the independence of the scientific advice received by the UK Government.

The regulations of medical practice are very clearly defined. Nevertheless, it appears that some crucial aspects of the medical profession, exercised through scientific advice, may not be accurately determined; thus revealing possible regulatory gaps. This vacuum seems to be more pronounced when a formal scientific advice is needed by the executive authority, designing the appropriate measures and strategies in the interests of the health of a nation.

Although, the specific advice offered to the UK Government may slip through the net of current regulations of the General Medical Council (GMC), it would be reasonable expecting that the advisors and advisory bodies to the Government would abide to the same rules followed by any clinician and researcher operating in the country. The concerns caused by the profoundly disturbing announcement of a herd immunity strategy in March 2020 (3) were worsened by the consideration that such medical strategy might have been shaped without peer review and adequate multidisciplinary input. This highly disputable decision supposedly was taken following the guidance of the Scientific Advisory Group for Emergency (SAGE). The legitimate concerns were accrued by the perceived lack of transparency as the members of the group remained secret for a considerable length of time, being publicly revealed only in April 2020 (4). Unsurprisingly, the quality of the scientific advice to the British Executive Authority has been openly criticized by numerous professionals holding international reputation; to the extent of being publicly challenged by the spontaneous constitution of an alternative and independent advisory group (5). Such events remain unique to the UK.

### Access to Hospital and Intensive Care

The analysis of the access to hospital and ICU has a pivotal importance in order to better understand the real impact that the COVID-19 had between February and July 2020 in the UK. Even though, almost every national healthcare providers have been admittedly overwhelmed by these unprecedented challenges, it has been suggested that this has not been the case for the NHS (6). For instance, in Italy, the Servizio Sanitario Nazionale (SSN) was clearly under remarkable strain despite a lower number of cases and more hospital beds per capita than UK (7, 8).

We have reviewed the SARS-CoV-2 report of the Intensive Care National Audit and Research Centre (ICNARC). The data presented by the ICNARC are highly reliable, following a rigorous and consolidated governance process (9). Our attention focused on demographic characteristics of COVID-19 patients, type of ventilatory support required on ICU admission, length of ICU stay, and final outcome. Given the fact that we could not find an equivalent source of information for national data as reliable as the ICNARC, with the aim of understanding whether our center would be comparable to UK average results, we decided to review the data from COVID-19 patients admitted to ICU at the Tor Vergata University Hospital (TVUH) in Rome, Italy (Table 1). This analysis showed that our COVID ICU had different patients' demographics and outcomes compared to UK averages. Specifically, the patients admitted to the TVUH COVID ICU appeared to be older and requiring more respiratory support on admission than their British counterpart. Probably, for such very reasons our patients might have suffered longer ICU hospitalisation associated with a higher mortality rate compared to those described in the ICNARC report. In this context, and bearing in mind the limitation of the above observations linked to different epidemiology, demographics, and healthcare organization, it may be highly relevant considering the activity of the NHS 111 telephone line that acted as “triage” system for patients with SARS-CoV-2 symptoms. It is rather worrying noticing that a number of concerns were raised regarding the process of clinical decisions. Such decisions have been leading to hospital admission or, conversely, to home management of subjects with documented symptomatic COVID-19. Such concerns are currently being investigated (10). Furthermore, it remains unclear how the status of “do not attempt resuscitation” applied to the elderly and the most vulnerable members of our society, might have affected their access to hospital care. Also, this issue is under investigation (11).

**Table 1:** Descriptive comparison (median with interquartile range or percentage) between Intensive Care National Audit and Research Centre (ICNARC) and Tor Vergata University Hospital (TVUH) data on SARS-CoV-2 patients admitted to intensive care unit (ICU) during the first wave of COVID-19 pandemic.

	**ICNARC**	**TVUH**
**Variables**	**Median (IQR) or %**	
Age (years)	60 (52-68)	69.5 (59-78)
**Outcome at end of ICU stay**		
Discharge Death	51.4 48.6	42.3 57.7
**Length of ICU stay (Days)**		
Survivor Non-Survivor	6 (3-13) 7 (4-13)	10 (5-28) 10 (1-33)
Mechanically ventilated within 24 h of ICU admission	65.7	100^*^

* *All patients were mechanically ventilated within 24 h of ICU admission according to ICNARC criteria: Intubated = 69.2%; BPAP = 30.8%*.

The process through which the access to hospital care is determined inevitably reflects on the overall mortality (12) and specifically to the data accuracy on the impact from COVID-19. Currently, there are two official sources of mortality data related to COVID-19 in the UK: The Department of Health and Social Care (DHSC) and The Office of National Statistics (ONS). The first institution reports all deaths occurring within 28 days of a positive test for SARS-CoV-2 whilst the second, a non-governmental authority, considers all deaths linked to SARS-CoV-2 as declared by the death certificates. Remarkably, the mortality rate presented by the ONS is about 20% higher than DHSC with an out of hospital mortality representing approximately 40% of the overall mortality (5, 13).

Certainly, providing accurate real-time data on the ongoing pandemic to the population and to professionals proved of being an immensely difficult task in any country. However, the discrepancy of the mortality rates between official institutions, inevitably, leads to subjective evaluation of the real impact of the pandemic in the UK.

### Non-pharmaceutical Intervention and Modelling

The announcement from pharmaceutical companies and some governments of the discovery of effective vaccines against SARS-CoV-2 has raised hopes of an imminent end of the pandemic (14). Certainly, the necessary scientific validation and the implementation of a global mass vaccination program will require time. As such, the recent discoveries have not diminished the value of NPI or the emphasis on reliable modelling to respond to potential second or third waves of COVID-19.

The effects of NPI aimed to contain the pandemic have been evaluated in a mathematical modelling (15). In this study, the adherence of the population to NPI has been briefly addressed. However, it deserves further discussion. Particularly, because it seems that the conclusions of the study have represented an important part of the scientific advice offered to the UK Government.

Demonstrably, adequate awareness leads to diligent adherence. This depends on the quality of the information divulged by public health officials, the scientific community, and MSM. This concept applies to many health conditions, as much as to the ongoing pandemic (16). The effects of NPI are strongly influenced by the adherence generated by the collective responsibility and public behavior (17). It has been reported that adherence to NPI during the COVID-19 pandemic varied substantially, depending on the single measure analyzed (18). It raises further concern the observation that a considerable portion of the population in the UK may not be prepared to follow simple basic NPI, such as social distancing and wearing a mask (19). Davies and colleagues, in their mathematical modelling, have assumed a compliance of 95% of all the British population (15). This estimate sounds over optimistic when compared to current evidence (20–22). Notably, it is not supported by any qualitative analysis neither any historical data endorse such extraordinarily high expected adherence. Instead, adherence is described in the appendix of the paper only as a “county to county” variation, with a regional compensation of adherence to NPI. It should be highlighted that an inferior adherence of only 1% of the population may actually involve more than half million UK citizens. Hence, reasonably questioning the conclusion of the study on number of cases, mortality, and resources of healthcare. It is highly relevant that the authors indicate that their analysis was part of the advice offered to the UK Government.

## Discussion

The extraordinary difficulties of shaping a response to the COVID-19 pandemic cannot be emphasised enough. The unprecedented medical and scientific challenges posed by an unknown virus have mercilessly exposed our vulnerabilities as individuals, together with the weaknesses of the healthcare services we dedicated our life. It is certainly strenuous identifying a country that flawlessly responded to SARS-CoV-2, conciliating the safeguard of the health of the nation with the scientific evidence and the inevitable increasing social pressures. On this regard, it is fundamental highlighting that major and even marginal socio-cultural and political differences between countries have substantially affected the governments responses as much as the compliance of populations. However, the peculiarities of the UK initial response to the pandemic deserve our attention for the consequences it had locally and outside the British borders.

It seems that the medical profession in the UK has witnessed during the first wave of COVID-19 what may be described as a continuous and progressive abandonment of the principles of best available evidence and safe practice, projected at national scale. Such withdrawal from the fundamental concepts of modern medicine, based on inclusiveness, multidisciplinary contribution, and transparency, has inevitably contributed to the highest mortality rate from SARS-CoV-2 in Europe, according to the ONS (5). The dereliction of clinical governance during the current healthcare crisis has implications beyond the tragic analysis we may perform today. Sadly, it represents a historical setback not only professionally, but also socially, contributing to solidarity failures (23).

Considering the magnitude of the professional advice to the Executive Authority, it would be appropriate that also the highest profile advice should follow the rigid processes of professional governance, in line with the processes that any individual clinician or institution regularly follows. Now more than ever, the GMC as a regulatory body independent from the Government and accountable to the Parliament may safeguard patients, doctors, and the health of the nation as stated by the GMC itself (24). The GMC could and should be involved by the UK Parliament to ascertain that “due diligence” has been applied to the process of advising the Government. Specifically, the GMC may be in the position to ensure that the principles of clinical governance would be applied to the whole process. This safety and governance processes may be implemented without interfering on the substance, merit, and confidentiality of the advice received by the Government. Unquestionably, the health of an entire country, as well as the credibility and public confidence on the medical profession have been put at risk. We all are conscious that Government policies may be disputed and opposed. It is inevitable and it does not represent a matter for our professional community. On the contrary, the professional advice to the Government from doctors registered in the GMC on public health issues of such relevance, must remain impeccable and untarnished. Certainly, the full understanding of the population on the magnitude of the pandemic has been influenced by the clarity of the information offered by the executive authorities, as much as their capacity of implementing restrictive measures.

A clear evaluation of hospital or ICU admission and related mortality between countries will be complex and lengthy. It will be even more difficult analyzing the out of hospital mortality, that in the UK it is particularly relevant. Also, attempting international comparisons would represent an extremely challenging task. Although our observation has numerous limitations, it is reasonable to postulate that the data from TVUH (one of the main COVID ICU of Central Italy) may actually reflect a national average; where the Northern regions were remarkably more afflicted by the pandemic compared to the Southern regions. The comparison between our local data and the report from ICNARC is merely indicative of possible different demographics and typology of admissions in the British ICUs. However, it certainly requires of being taken into account when an accurate assessment with a rigorous multivariate statistical model in the context of a properly designed study will be performed. An in-depth analysis including serological estimates in relation to hospitalizations and ICU admissions (25) will remain scientifically and socially necessary in order to better understand the evolution of the pandemic in the UK and elsewhere; thus implementing the adequate corrections to the healthcare services and increasing the compliance of the population to new stringent measures aimed to control further waves of the pandemic. More importantly, it would be a valid reassurance for the British population, clarifying whether any selection bias has been applied to prevent the overwhelming of the NHS as it seems that might have happened (26).

The behavior of the population is of extraordinary relevance in modelling the actual response to a healthcare crisis of the proportion of the COVID-19 pandemic. Including adherence variation in a mathematical modelling may be complex but crucially important. Undoubtedly, the level of health education of the population, associated with the level of trust on professional or institutional advice, have played an important role on the adherence to NPI across regions of the same country and between different nations. Therefore, considering parameters predictive of behavior of the population such as awareness, isolation fatigue, and trust will be required in order to corroborate the prediction of the effects of each NPI. In fact, stratifying the expected adherence to specific NPI will enhance the reliability of mathematical modelling. Including also realistic adherence variables will contribute to shape effective strategies and efficient response at both national and regional level (27–29).

The awareness on the risks and effects of SARS-CoV-2 and consequently the adherence to the NPI is jeopardized by the presentation of dubious information such as those on mortality rate. Haphazardly, the general public in the UK has been left building its own knowledge on the impact of the pandemic, navigating between complacent official reports and tragically correct non-governmental data (5, 13). It would have been certainly beneficial if governments and MSM could have been referring to a much stronger guidance or code of conduct by the WHO on data analysis and a clearer standardized public presentation of the COVID-19 scenario.

**Figure 1:**
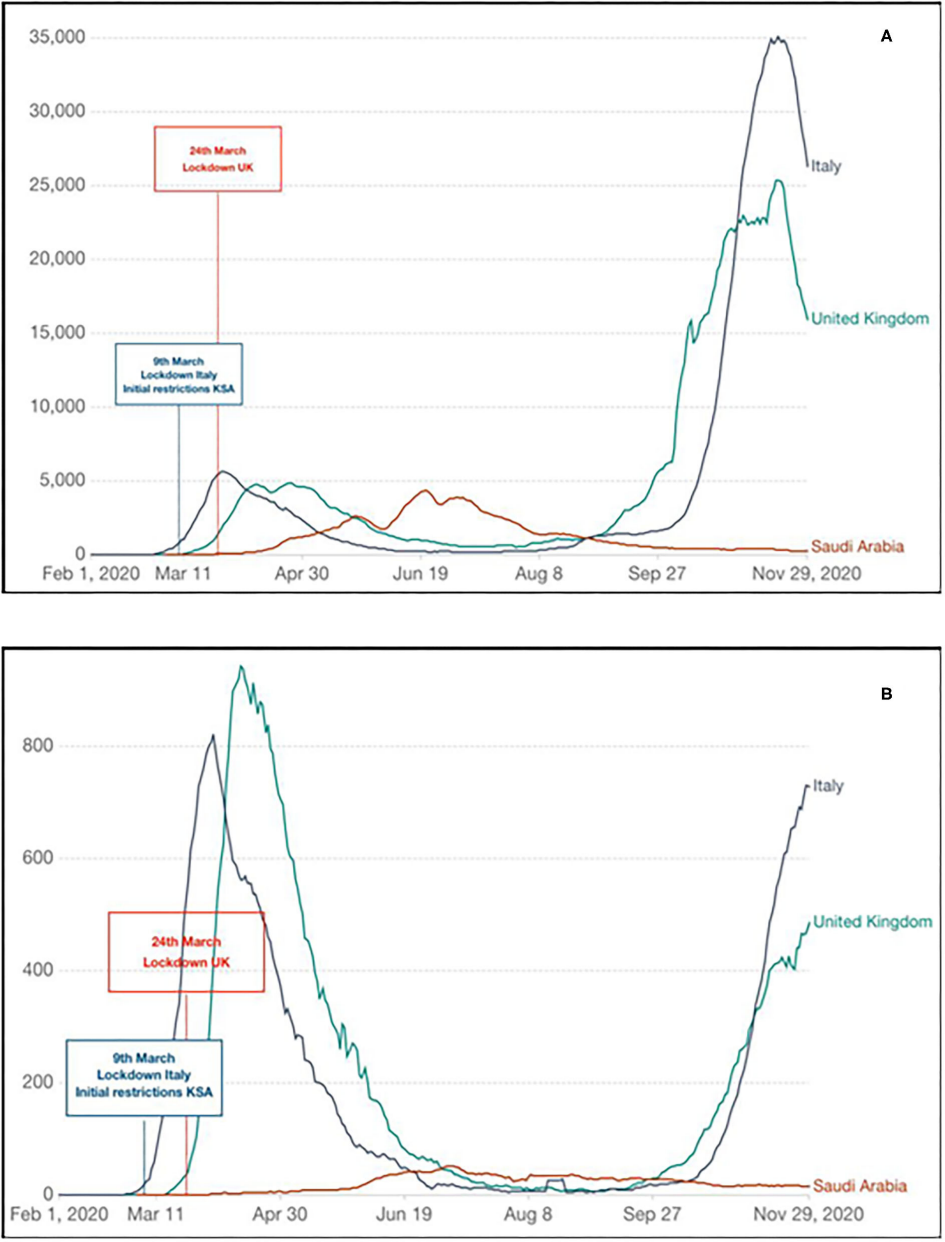
Evolution of COVID-19 first wave of pandemic in the United Kingdom (UK), Italy, and Kingdom of Saudi Arabia (KSA) indicating timing of initial response and impact: **(A)** new SARS-CoV-2 confirmed cases (seven rolling days average); **(B)** new SARS-CoV-2 confirmed deaths (seven rolling days average). Diagrams generated and adapted from Our World in Data (https://ourworldindata.org); Data source: CDC Europe (https://www.ecdc.europa.eu/en).

A strong indicator of the benefit arising from prompt implementation and diligent use of NPI, associated with consistent and uncompromising information to the population, was observed in the Kingdom of Saudi Arabia (KSA) (30, 31). In the KSA, there was a gradual introduction of restrictions since the very early stages of the first wave of pandemic (6th March 2020), despite a limited number of cases, regionally confined. In this country, a second wave of SARS-CoV-2 was observed much earlier than Western Europe. It followed the Holy Month of Ramadan, coinciding with the easing of some restrictions and domestic flights resume on 31st May 2020 (32). The remarkable quick response of the Government linked with an excellent compliance to NPI has undoubtedly contributed to delay the first wave of pandemic; subsequently controlling the second wave effectively in less than two months, without reimposing strict public health measures. The national KSA strategy has also been rewarded with a lower incidence of cases and mortality as indicated in Figures 1A,B, respectively. Other countries, following the same principles, have succeeded in limiting the impact of COVID-19, New Zealand and South Korea are the most cited examples (33, 34).

Although the consideration on the implication of adherence in the study of Davies et al. may be of interest and debated, unequivocally, the authors indicate in their conclusions that the executive authority in the UK was fully aware of the risk posed by SARS-CoV-2, as much as of the unacceptable expected mortality of a “mitigation strategy”, very well before the UK lockdown date on 24th March 2020 (14). Crucially, it should be noted that on the 6th of March 2020, in Italy thee was an average of 530 cases a week with 25 weekly deaths reported. In the UK, on the same date, there were 18 cases and no deaths reported. In the KSA, there were 17 cases and zero reported deaths on 16th March, which is a week after the NPI measures were implemented incrementally, reaching a complete lockdown with 24 h curfew on 9th April.

Our critical analysis focuses on some relevant aspects of the initial UK response to the COVID-19 pandemic that have not been properly addressed despite being at the very core of highly controversial events and adverse outcomes. In particular, messaging variables and constituent response, lack of transparency on scientific advices and political choices associated with misinformation regarding the magnitude of the pandemic and the actual resources of the national healthcare provider, deserve scientific attention. In an attempt to support our considerations improving the clarity of the message delivered, we arbitrarily decided to compare specific elements of the early British response to those of the KSA and Italy. Certainly, it may be argued that other countries could have been used for comparison. In this regard, the most frequent terms of comparison presented by MSM and professional publications have been South Korea and New Zealand that proved particularly successful in managing the first wave of pandemic. However, the two examples we made, regarding the typology of ICU patients in Italy and promptness of the response in the KSA, represent in our opinion a very pertinent choice as they gave us to the opportunity to highlight and explain specific and remarkable differences without necessarily attempting a formal comparison on all aspects of the response to SARS-CoV-2. Importantly, we decided to restrict the analysis to those specific nations reflected by our affiliations and from where we could obtain meaningful comparable data. At present, there are very limited number of national studies and/or data sources describing the typology of patients admitted to COVID ICU and their course during COVID ICU stay. Therefore, we have chosen to use the most reliable information we could obtain comparing data extracted from the ICNARC report with the ones directly collected from our COVID ICU in Italy. On the other hand, the KSA data were analysed because of the striking difference with the UK in establishing the initial response. In fact, in the KSA the timing of the COVID response has mirrored the implementation of countrywide restrictions in some European Countries including Italy; this was despite a lower number of cases compared to the UK. As mentioned above, such observations and critical analysis were naturally done also because of the affiliations of the authors.

While remarking the perspective and narrative nature of our analysis, defending the genuine choices we made constructing it, we recognise that its greatest limitation is the lack of a formal discussion and in-depth analysis of the socio-cultural and political variables that distinguish the UK, Italy, and the KSA. Reasonably, such differences may represent a significant bias as they affect the strength of the restrictive measures endorsed by the authorities, the rights and freedom of the populations involved to criticise and resist the governments' choices, the way the pandemic-related messaging is conceptualised, packaged, and presented to the citizens, and the actual possibility of the people to understand and copy with scientific and technical information, as well as their ability to adhere to NPI. Nevertheless, addressing these elements in this specific context would be extremely challenging and perhaps outside the primary objective of our considerations.

It may be strongly argued that the UK has suffered the highest mortality rate in Europe from the first wave of COVID-19 following a delayed response in implementing the adequate measures, despite witnessing the tragic evolution of the pandemic in other countries such as Italy and Spain. The public divulgation of the impact of a “mitigation strategy” on 16th March 2020 (35) has certainly contributed to a sudden change of direction of the British strategy. The modalities of such divulgation would deserve further reflection as to whether these modalities may reflect more a sense of urgency from a member of the SAGE rather than an academic contribution “per se” (35).

In the UK, beyond the organizational and medical complexities of the management of SARS-CoV-2, unique events influencing the scientific analysis and medical advice to the Government, the access to hospital care, and the implementation of the necessary NPI have affected the health of a nation. Sadly, it would suggest that in current extraordinary times, the “*Salus populi*” may not be a “*suprema lex*”.

## Data Availability Statement

The raw data supporting the conclusions of this article will be made available by the authors, without undue reservation.

## Ethics Statement

Ethical review and approval was not required for the study on human participants in accordance with the local legislation and institutional requirements. The patients/participants provided their written informed consent to participate in this study.

## Author Contributions

EF and RC: conceptualization and writing—original draft preparation. FL, TM, RA, and YA: data collection. FL, TM, RA, and YA: data analysis. EF, RC, and CA: data interpretation. EF, RC, TM, RA, YA, and CA: literature review. EF, RC, and FL: writing—review and editing. RC and YA: supervision. All authors contributed to the article and approved the submitted version.

## Conflict of Interest

The authors declare that the research was conducted in the absence of any commercial or financial relationships that could be construed as a potential conflict of interest.

## Publisher's Note

All claims expressed in this article are solely those of the authors and do not necessarily represent those of their affiliated organizations, or those of the publisher, the editors and the reviewers. Any product that may be evaluated in this article, or claim that may be made by its manufacturer, is not guaranteed or endorsed by the publisher.
